# Correlation of the triglyceride-glucose-body mass index with all-cause and cardiovascular mortality in patients undergoing peritoneal dialysis: a retrospective cohort study

**DOI:** 10.3389/fnut.2025.1612402

**Published:** 2025-08-20

**Authors:** Jinping Li, Xichao Wang, Wenyu Zhang, Na Sun, Yingying Han, Wenxiu Chang

**Affiliations:** Department of Nephrology, Tianjin First Central Hospital, Tianjin, China

**Keywords:** triglyceride-glucose-body mass index, peritoneal dialysis, all-cause mortality, cardiovascular mortality, insulin resistance

## Abstract

**Background:**

The triglyceride-glucose-body mass index (TyG–BMI) is a simple indicator of insulin resistance and is linked to an elevated risk of mortality. Nevertheless, limited research has explored the associations between the TyG–BMI and all-cause and cardiovascular mortality in patients undergoing peritoneal dialysis (PD).

**Methods:**

Patients initiating PD treatment at the Tianjin First Central Hospital’s Nephrology Department from July 2013 to February 2024 had triglycerides, fasting blood glucose, height, and weight measured at baseline and monthly during follow-up. TyG–BMI was calculated, dividing PD patients into high, middle, or low TyG–BMI groups using the tri-quantile method. Cox regression analysis assessed hazard ratios (HRs) for all-cause and cardiovascular mortality among these groups. A restricted cubic spline regression was used to explore the relationship between TyG–BMI and the primary and secondary outcomes.

**Results:**

A total of 865 patients were included. The mean TyG–BMI value for the entire study population was 212.27 ± 46.64. Patients in the high TyG–BMI group had a higher proportion of patients whose primary kidney disease was diabetic nephropathy and the greatest proportion of patients with comorbid diabetes mellitus. During the follow-up, 266 (30.75%) deaths occurred, with CVD being the dominant cause in 110 (41.35%) patients. Univariate and multivariate Cox regression analyses showed that middle group patients had a significantly lower risk of all-cause mortality compared to other groups. For CVD mortality, high group patients had a significantly greater hazard ratio than middle group patients, while there was no significant difference between the low and middle groups. Restricted cubic spline regression revealed a U-shaped association between TyG–BMI and all-cause mortality risk, as well as a J-shaped association with CVD mortality; inflection points were identified at 209.73 and 206.64, respectively. In the subgroup analysis, we found that higher TyG–BMI values were associated with increased all-cause and cardiovascular mortality in men, and lower TyG–BMI values were linked to elevated all-cause mortality in women.

**Conclusion:**

The TyG–BMI shows U-shaped and J-shaped relationships with all-cause and CVD mortality risk, respectively, in PD patients. Additionally, significant sex differences were observed in these associations.

## Background

Chronic kidney disease (CKD) has emerged as a significant global public health crisis, characterized by a steadily increasing prevalence rate of approximately 10%. CKD is recognized not only as a direct contributor to global morbidity and mortality but also as a substantial risk factor for cardiovascular disease (CVD) (1–6). The prevalence of CVD among patients with CKD is markedly higher than that observed in the general population. Furthermore, the incidence of CVD exhibits an inverse correlation with renal function (7); it remains the leading cause of mortality in patients undergoing peritoneal dialysis (PD) (8). PD is a primary therapeutic modality for treating end-stage renal disease. The technical simplicity, reduced need for trained staff, lower nurse-to-patient ratios, cost-effectiveness, and other factors have led to the implementation of the PD-First policy over several decades (9–13). CKD, type 2 diabetes (T2D), and CVD are interconnected and mutually influential, with an increasing body of evidence demonstrating their systemic interdependence. This has led to the emergence of the term cardio-metabolic-renal (CMR) disease (14). Insulin resistance (IR) may play a significant role in this context. PD initially improves IR in uremic patients (15); however, concerns arise regarding potential long-term effects such as systemic hyperglycemia, obesity, and aggravated IR due to cumulative exposure to glucose solutions used in PD (peritoneal glucose exposure, advanced glycation end products, and bioincompatible solutions), which could contribute to increased cardiovascular risk (16, 17).

The triglyceride-glucose-body mass index (TyG–BMI), an alternative IR index independent of insulin, was identified as an independent risk factor for diabetic kidney disease (DKD) (18). Recent studies have demonstrated that the TyG–BMI is associated with the risk of a composite outcome including acute myocardial infarction, repeat revascularization, stroke, hypertension, coronary artery disease, and all-cause mortality (19–22). Nevertheless, the associations between the TyG–BMI and all-cause as well as CVD mortality in the PD population of Chinese origin remain to be elucidated. Hence, this study was conducted to explore this relationship within a single-center cohort of the Chinese PD population.

## Materials and methods

### Study population

We retrospectively established a PD cohort between July 2013 and February 2024 at the Department of Nephrology, Tianjin First Central Hospital, China. Patients aged 18–80 years who initiated PD treatment for at least 3 months were enrolled in this study. All patients were treated according to the high-quality goal-directed peritoneal dialysis strategy of the International Society for Peritoneal Dialysis Practice Recommendations at our hospital before enrollment and during the observation period. Patients were excluded if they had malignant tumors, were receiving chemotherapy, or had insufficient clinical information. Patients who were transferred to another hospital, switched to hemodialysis, or received a kidney transplant during the study period were treated as censored and included in the survival analysis. This study has been granted an exemption from requiring written informed consent because it is a retrospective review of medical records. Prior to analysis, the patient records and information were anonymized and de-identified. This study was conducted at the PD Center of the Nephrology Department at our hospital, approved by the institutional review board (IRB) of the hospital (2015009S), and was conducted in accordance with the principles of the Helsinki Declaration.

### Data collection and definitions

Data were obtained from medical records. Information was collected on sociodemographic characteristics, medical history, anthropometric measurements, and laboratory tests. PD patients in our center underwent monthly hematological and biochemistry examinations during their outpatient follow-up.

Baseline demographic and clinical information, including age, sex, original disease, height, weight, systolic blood pressure (SBP), diastolic blood pressure (DBP), diabetes mellitus (DM), hypertension history, and body mass index (BMI), was collected at the initiation of PD. SBP and DBP were measured in the morning of the outpatient visits. Diabetes was defined as a self-reported history of diabetes or FBG ≥ 7.0 mmol/L. Hypertension was defined as a self-reported history of hypertension, the use of antihypertensive medication, SBP ≥ 140 mmHg, or DBP ≥ 90 mmHg. Hypercholesterolemia was defined as a self-reported history of dyslipidemia, or TC ≥ 5.17 mmol/L. Body mass index (BMI) was calculated as weight (kg)/height (m)^2^.

Biochemical and medication data were collected 1 month after PD onset as a baseline. Blood parameters included hemoglobin (Hb), albumin (Alb), blood urea nitrogen (BUN), creatinine (Cr), uric acid (UA), sodium (Na), potassium (K), chlorine (Cl), venous carbon dioxide (CO_2_), calcium (Ca), inorganic phosphorus (P), fasting blood glucose (FBG), triglyceride, total cholesterol (TC), high-density lipoprotein cholesterol (HDL-C), low-density lipoprotein cholesterol (LDL-C), high-sensitivity C-reactive protein (hsCRP), and intact parathyroid hormone (iPTH). All blood samples were measured and analyzed via commercially available kits and an automated analyzer (Cobas 8000 or e411 for iPTH, Roche Diagnostics Ltd. for others, Germany). The serum albumin concentration was measured via the bromocresol green method. The average 24-h urinary urea and creatinine clearances were calculated to assess residual renal function (RRF). The total PD treatment volume, total ultrafiltration volume, total effluent dialysate volume, and daily urine volume were recorded daily. The total protein content in the effluent dialysate was measured. The protein equivalent nitrogen appearance (nPNA) was calculated and normalized to the actual weight. In accordance with previous studies (23–27), the TyG index was calculated as Ln [TG (mg/dL) × FBG (mg/dL)/2]. TyG–BMI = TyG × BMI (22). Treatment with angiotensin-converting enzyme inhibitors or angiotensin II receptor blockers (both combined as renin-angiotensin-aldosterone system (RAAS) inhibitors, RAASi) was also recorded. PD types and associated parameters were recorded. PD patients received standard prescriptions for continuous ambulatory peritoneal dialysis (CAPD using Baxter’s Low Calcium Peritoneal Dialysis Solution [Lactate-G1.5/2.5%]) or automated peritoneal dialysis (APD using Baxter’s Peritoneal Dialysis Solution [Lactate-G1.5/2.5%]), accordingly. Twenty-four-hour urine and dialysate samples were collected 1 month after PD commencement and every month thereafter to measure dialysis adequacy, which was defined as the urea clearance index (Kt/V) and creatinine clearance (CCr), via standard methods (28). Weekly CCr (WCCr) was calculated by multiplying the 24-h Ccr by 7 days.

### Study outcomes

The primary and secondary endpoints were all-cause mortality and CVD mortality, respectively. The criteria for CVD death were death attributed to congestive heart failure, acute myocardial infarction, atherosclerotic heart disease, cardiac arrest, cardiac arrhythmia, cardiomyopathy, cerebrovascular disorders, anoxic encephalopathy, ischemic brain injury, or peripheral arterial disease (29). The cause of death was identified by a comprehensive management team consisting of junior and senior professors at our PD Center.

All patients were followed up until death, transferred to hemodialysis therapy, received a kidney transplant, transferred to another center, lost to follow-up, or until 29 February 2024.

### Statistical analysis

Normally distributed continuous variables are presented as means±standard deviations (SDs), while non-normally distributed continuous variables are presented as medians and interquartile ranges. Categorical variables are presented as frequencies and percentages. All patients were divided into three groups according to TyG–BMI values using the tri-quantile method: the high group (TyG–BMI>227.15), middle group (TyG–BMI 189.57–227.15), and low group (TyG–BMI < 189.57). Comparisons of continuous variables among the three groups were performed using a one-way ANOVA or the Kruskal–Wallis H test. The chi-squared test was used to compare categorical variables. Survival analysis was performed using Cox regression to compare the effects on all-cause and cardiovascular mortality among the high, middle, and low groups. A restricted cubic spline plot with five knots (at the 5th, 27.5th, 50th, 72.5th, and 95th percentiles) was used to explore the relationship between TyG–BMI, as a continuous variable, and the outcomes. Model 1 was univariate. Model 2 was adjusted for sex, age, original disease, DM, hypertension, and SBP. Model 3 was additionally adjusted for Hb, Alb, K, CO_2_, P, total Kt/V urea, nPNA, RRF, iPTH, and RAASi. Survival curves were plotted using the Kaplan–Meier method, and comparisons between groups were performed using the log-rank test. All statistical analyses were conducted using SPSS version 22 (IBM, Japan) or the statistical package R (version 4.5.1). A *p*-value of less than 0.05 was considered statistically significant.

## Results

### Clinical characteristics and laboratory data among the three TyG–BMI groups

**Figure 1:**
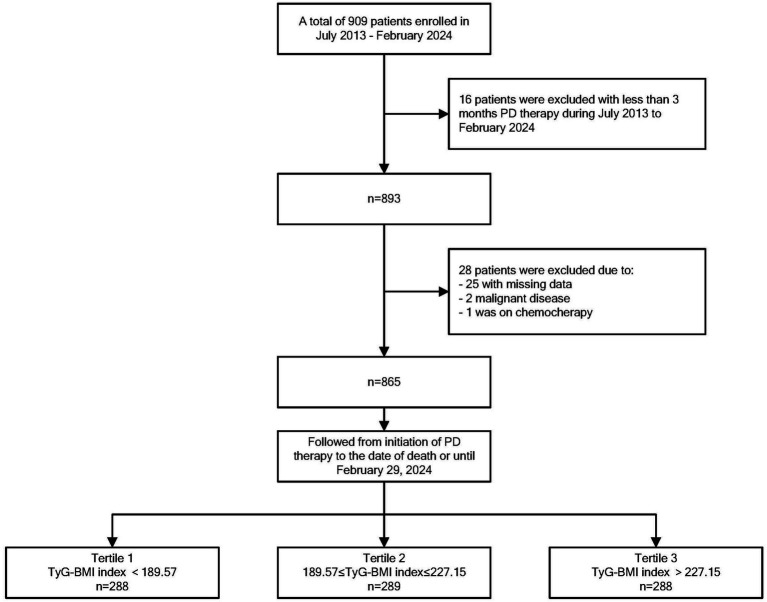
Flowchart of patient selection.

In this study, we excluded patients with missing data (*n* = 25), patients with malignant disease (*n* = 2), and patients who received chemotherapy during the follow-up period (*n* = 1). Therefore, 865 eligible patients were included in the current study (Figure 1). The mean age of the 865 participants was 57.28 ± 15.27 years, and 480 (55.5%) of them were male. The mean TyG–BMI of the entire study population was 212.27 ± 46.64. The clinical characteristics and laboratory data of the three groups categorized by the TyG–BMI are shown in Table 1. Patients in the high TyG–BMI group had the lowest prevalence of glomerulonephritis as the primary disease, the highest prevalence of diabetic nephropathy and hypertensive nephropathy, and the greatest number of individuals with diabetes. They also had the highest mean SBP, triglyceride, BMI, Hb, and hs-CRP levels, as well as the lowest mean total cholesterol level. In the middle group, patients were more likely to be male and had the lowest prevalence of hypertensive nephropathy as the original disease. These patients also exhibited the lowest mean triglyceride levels and the highest HDL-C levels. Patients in the low group exhibited the highest prevalence of glomerulonephritis as the original disease and the lowest prevalence of diabetic nephropathy. This group also had the fewest individuals diagnosed with diabetes. Furthermore, they presented with the lowest mean levels of SBP, BMI, Hb, HDL-C, and hs-CRP, along with the highest mean total cholesterol level.

**Table 1:** Clinical characteristics and laboratory data among the three TyG–BMI groups.

Characteristic	Total (*n* = 865)	High group (*n* = 288)	Middle group (*n* = 289)	Low group (*n* = 288)	*p*-value^*^
Age (y)	57.28 ± 15.27	56.64 ± 14.00	58.26 ± 14.39	56.88 ± 17.27	0.381
Sex (male, %)	480 (55.5%)	165 (57.3%)	173 (59.8%)	142 (49.3%)	0.031
Original disease					<0.001
Glomerulonephritis	367 (42.4%)	93 (32.3%)	115 (39.9%)	159 (55.2%)	
Diabetic nephropathy	267 (30.9%)	126 (44.0%)	99 (34.2%)	42 (14.6%)	
Hypertensive nephropathy	87 (10.1%)	34 (11.7%)	24 (8.3%)	29 (10.1%)	
Others	144 (16.6%)	35 (12.1%)	51 (17.6%)	58 (20.1%)	
DM (%)	300 (34.7%)	142 (49.3%)	108 (37.5%)	50 (17.4%)	<0.001
Hypertension (%)	430 (49.7%)	142 (49.3%)	155 (53.8%)	133 (46.2%)	0.147
SBP (mmHg)	150.51 ± 20.76	154.30 ± 20.41	150.39 ± 20.31	146.86 ± 20.98	<0.001
BMI (kg/m^2^)	20.77 ± 9.22	24.85 ± 10.35	20.43 ± 8.20	17.06 ± 7.19	<0.001
TyG–BMI	212.27 ± 46.64	263.88 ± 37.17	207.72 ± 10.83	165.52 ± 17.12	<0.001
Blood parameters					
Hb (g/L)	84.21 ± 17.60	87.66 ± 17.48	83.89 ± 17.05	81.11 ± 17.75	<0.001
Alb (g/L)	34.94 ± 5.87	35.02 ± 5.57	34.76 ± 5.96	35.04 ± 6.09	0.813
BUN (mmol/L)	25.06 ± 11.83	24.62 ± 10.84	24.53 ± 10.88	26.08 ± 13.59	0.214
Cr (μmol/L)	725.40 ± 297.22	717.39 ± 262.17	723.01 ± 289.94	735.94 ± 336.08	0.749
UA (μmol/L)	409.17 ± 153.57	422.77 ± 145.81	406.76 ± 149.86	398.14 ± 164.17	0.154
Na (mmol/L)	139.16 ± 4.29	139.17 ± 3.96	138.93 ± 4.60	139.40 ± 4.28	0.434
K (mmol/L)	4.58 ± 0.84	4.56 ± 0.84	4.61 ± 0.87	4.58 ± 0.82	0.763
Cl (mmol/L)	102.79 ± 4.07	104.95 ± 9.09	101.85 ± 5.14	101.65 ± 7.14	0.433
CO_2_ (mmol/L)	20.72 ± 4.57	20.44 ± 4.53	20.83 ± 4.51	20.87 ± 4.66	0.464
Ca (mmol/L)	1.93 ± 0.34	1.93 ± 0.28	1.95 ± 0.44	1.92 ± 0.2	0.549
P (mmol/L)	1.85 ± 0.62	1.90 ± 0.64	1.81 ± 0.53	1.84 ± 0.68	0.255
FBG (mg/dL)	114.15 ± 48.33	127.99 ± 52.64	111.68 ± 48.09	102.94 ± 40.21	0.099
Triglyceride (mg/dL)	1.85 ± 0.62	1.90 ± 0.64	1.81 ± 0.53	1.84 ± 0.68	0.033
Total cholesterol (mmol/L)	0.92 ± 0.19	0.90 ± 0.18	0.93 ± 0.18	0.94 ± 0.20	0.001
HDL-C (mmol/L)	43.15 ± 14.19	44.71 ± 19.49	45.48 ± 17.10	39.03 ± 14.14	<0.001
LDL-C (mmol/L)	156.47 ± 100.68	207.13 ± 127.19	148.63 ± 79.71	114.03 ± 61.18	0.244
hs-CRP (mg/L)	4.36 [1.98, 10.70]	4.87 [2.26, 11.55]	4.21 [1.99, 11.30]	3.59 [1.82, 9.49]	<0.001
iPTH (pg/mL)	289.00 [175.25, 451.28]	291.80 [190.70, 442.20]	293.20 [175.50, 450.20]	267.20 [163.30, 459.40]	0.509
Treatment					
RAASi (%)	532 (61.5%)	172 (59.7%)	176 (60.9%)	184 (63.9%)	0.376

DM, diabetes mellitus; SBP, systolic blood pressure; BMI, body mass index; TyG–BMI, triglyceride glucose-body mass index; Hb, hemoglobin; Alb, albumin; BUN, blood urea nitrogen; Cr, creatinine; UA, uric acid; Na, sodium; K, potassium; Cl, chlorine; CO_2_, venous carbon dioxide; Ca, calcium; P, phosphorus; FBG, fasting blood glucose; HDL-C, high-density lipoprotein cholesterol; LDL-C, low-density lipoprotein cholesterol; hs-CRP, high-sensitivity C-reactive protein; iPTH, intact parathyroid hormone; RAASi, RAAS inhibitor.

*The ANOVA test, Kruskal–Wallis H test (hsCRP, iPTH), or chi-squared test as appropriate.

### Peritoneal dialysis-related parameters among the three TyG–BMI groups

As shown in Table 2, the majority of PD patients in this cohort used the daytime automated peritoneal dialysis (DAPD) modality (80.6%), and the median RRF was 2.8 mL/min/1.73m^2^, the urine volume was 800 mL/day, and the nPNA was 0.93 g/kg/d. The mean total Kt/V was 1.96 ± 0.84, and the total WCCr was 70.81 ± 45.20 L/w/1.73m^2^. Patients in the High TyG–BMI group had the highest RRF, urine volume, total WCCr, and renal WCCr, and the lowest total Kt/V, renal Kt/V, dialysate Kt/V, and nPNA. In the middle group, patients had the lowest RRF. Patients in the low group exhibited the lowest urine volume, total WCCr, and renal WCCr, while demonstrating the highest values for total Kt/V, renal Kt/V, dialysate Kt/V, and nPNA.

**Table 2:** Peritoneal dialysis-related parameters among the three TyG–BMI groups.

Characteristics	Total (*n* = 865)	High group (*n* = 288)	Middle group (*n* = 289)	Low group (*n* = 288)	*p*-value^*^
PD type					0.386
CAPD (%)	128 (14.8%)	46 (16.0%)	44 (15.3%)	38 (13.2%)	
APD (%)	40 (4.6%)	18 (6.4%)	12 (4.0%)	10 (3.5%)	
DAPD (%)	697 (80.6%)	224 (77.7%)	233 (80.7%)	240 (83.3%)	
RRF (mL/min/1.73m^2^)	2.80 [0.67, 5.07]	3.85 [1.08, 6.41]	2.47 [0.51, 4.54]	2.58 [0.58, 4.32]	0.001
Urine volume (mL/day)	800.00 [500.00, 1300.00]	1000.00 [500.00, 1500.00]	885.00 [500.00, 1300.00]	715.00 [487.5, 1100.00]	<0.001
Total Kt/V	1.96 ± 0.84	1.80 ± 0.53	1.91 ± 0.61	2.17 ± 1.20	<0.001
Renal Kt/V	1.06 ± 0.52	1.01 ± 0.43	1.04 ± 0.55	1.14 ± 0.55	0.007
Dialysate Kt/V	0.91 ± 0.84	0.81 ± 0.46	0.89 ± 0.59	1.04 ± 0.86	0.007
Total WCCr (L/w/1.73m^2^)	70.81 ± 45.20	77.21 ± 29.91	68.52 ± 32.40	66.80 ± 35.29	0.017
Renal WCCr (L/w/1.73m^2^)	40.70 ± 29.83	48.76 ± 31.52	39.06 ± 29.31	34.33 ± 26.74	<0.001
Dialysate WCCr (L/w/1.73m^2^)	30.27 ± 38.72	28.95 ± 15.25	29.42 ± 19.36	32.53 ± 23.45	0.512
nPNA (g/kg/d)	0.93 [0.79, 1.16]	0.86 [0.73, 1.03]	0.89 [0.76, 1.10]	1.02 [0.86, 1.23]	<0.001

CAPD, continuous ambulatory peritoneal dialysis; APD, automated peritoneal dialysis; DAPD, automated peritoneal dialysis at daytime; RRF, residual renal function; Kt/V, urea clearance index; WCCr, weekly creatinine clearance; nPNA, the normalized protein equivalent of total nitrogen appearance.

* The ANOVA test, Kruskal–Wallis H test (RRF, urine volume, nPNA), or chi-squared test, as appropriate.

### Cox regression models for the all-cause and CVD mortality

During the 46.6 (22.4–78.0) months of follow-up, 266 (30.75%) deaths occurred, of which 110 (41.35%) were due to CVD. When analyzed as a continuous variable for Cox regression, TyG–BMI did not affect all-cause mortality. Categorically, patients in the high and low groups were significantly associated with an increased risk of all-cause mortality compared to those in the middle group according to the univariate and multivariate analyses (Table 3). TyG–BMI was significantly associated with CVD mortality, which was analyzed as a continuous variable in all three models. Only patients in the high TyG–BMI group had an increased risk of CVD mortality compared to those in the middle group. There was no significant difference between the low group and the middle group (Table 4).

**Table 3:** Cox regression analysis for the effects of the TyG–BMI and TyG–BMI categorical groups on all-cause mortality (*n* = 865).

Parameter	Model 1			Model 2			Model 3		
	HR	95% CI	*p*-value	HR	95% CI	*p*-value	HR	95% CI	*p*-value
TyG–BMI	1.00	1.00 to 1.01	0.100	1.00	1.00 to 1.01	0.859	1.00	1.00 to 1.01	0.318
Categorical groups									
High group vs. middle group	1.58	1.17 to 2.14	0.003	1.47	1.08 to 2.00	0.014	1.84	1.03 to 3.27	0.038
Low group vs. middle group	1.54	1.13 to 2.09	0.006	1.67	1.21 to 2.30	0.002	1.92	1.10 to 3.35	0.022

TyG–BMI, triglyceride-glucose-body mass index.

Model 1: Univariate.

Model 2: Adjusted for sex, age, original disease, DM, hypertension, and SBP.

Model 3: Adjusted for sex, age, original disease, DM, hypertension, SBP, Hb, Alb, K, CO_2_, P, total Kt/V urea, nPNA, RRF, iPTH, and RAASi.

**Table 4:** Cox regression analysis for the effects of the TyG–BMI and TyG–BMI categorical groups on cardiovascular mortality (*n* = 865).

Parameter	Model 1			Model 2			Model 3		
	HR	95% CI	*p*-value	HR	95% CI	*p*-value	HR	95% CI	*p*-value
TyG–BMI	1.01	1.00 to 1.01	0.001	1.01	1.00 to 1.01	0.049	1.01	1.00 to 1.02	0.015
Categorical groups									
High group vs. middle group	2.04	1.28 to 3.27	0.003	1.89	1.17 to 3.06	0.009	2.94	1.20 to 7.21	0.019
Low group vs. middle group	1.46	0.88 to 2.41	0.145	1.68	0.99 to 2.85	0.053	2.06	0.74 to 5.70	0.166

TyG–BMI, triglyceride-glucose-body mass index.

Model 1: Univariate.

Model 2: Adjusted for sex, age, original disease, DM, hypertension, and SBP.

Model 3: Adjusted for sex, age, original disease, DM, hypertension, SBP, Hb, Alb, K, CO_2_, P, total Kt/V urea, nPNA, RRF, iPTH, and RAASi.

### Kaplan–Meier analysis and log-rank test

**Figure 2:**
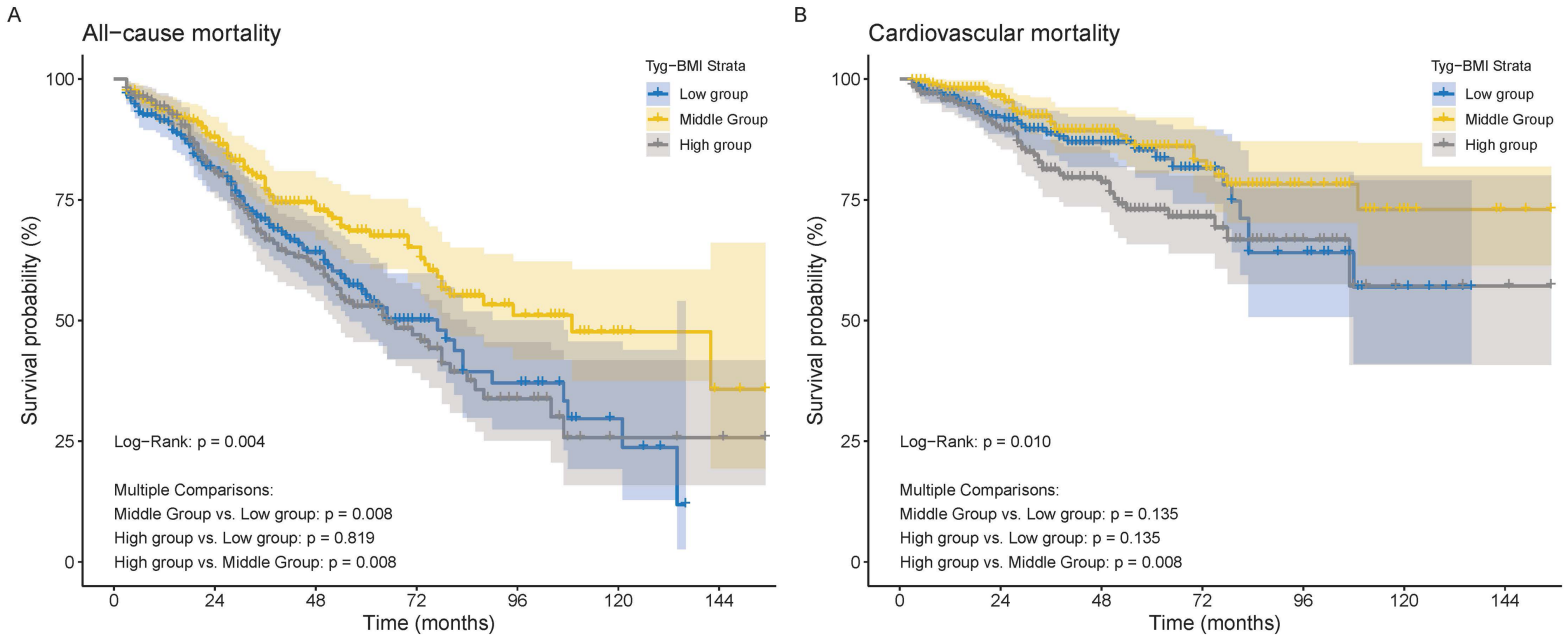
The Kaplan–Meier survival analysis curves for mortality. **(A)** All−cause mortality. **(B)** Cardiovascular mortality.

The Kaplan–Meier analysis and the log-rank test were performed among the three groups, with survival curves illustrated in Figure 2. Regarding all-cause mortality, a statistically significant difference was observed among the three groups (*p* = 0.0043) (Figure 2A). In the context of multiple comparisons, patients in both the high and low groups demonstrated a significantly increased risk of mortality when compared to those in the middle group. There was also a statistically significant difference in CVD mortality among the three groups (*p* = 0.0098) (Figure 2B). In the context of multiple comparisons, patients classified in the high group exhibited a significantly elevated risk of CVD mortality when compared to those in the middle group (*p* = 0.008).

**Figure 3:**
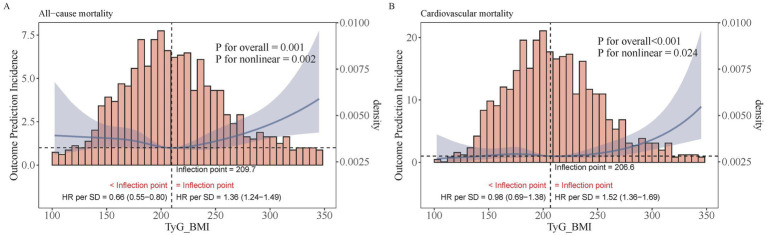
Restricted cubic regression between TyG–BMI and mortality. **(A)** All−cause mortality. **(B)** Cardiovascular mortality.

Restricted cubic spline regression analysis revealed a significant U-shaped relationship between TyG–BMI and all-cause mortality. The risk of death was found to be lowest at a TyG–BMI value of 209.73, with an increasing trend observed both before and after this point (p for non-linearity = 0.002). For TyG–BMI < 209.73, the HR increases by 0.66 times for every one standard deviation decrease (95% CI [0.55–0.80]), whereas for TyG–BMI > 209.73, the HR increases by 1.36 times for every one standard deviation increase (95% CI [1.24–1.49]) (Figure 3A). No similar relationship was evident between TyG–BMI and cardiovascular mortality; instead, a J-shaped pattern was observed (non-linear *p* = 0.024). The risk of CVD mortality remains relatively stable until TyG–BMI reaches 206.64, after which it begins to increase rapidly (non-linear *p* = 0.024). When TyG–BMI is < 206.64, there is no statistically significant difference in HR for every one standard deviation reduction (95% CI [0.69–1.38]); when TyG–BMI is > 206.64, the risk increases 1.52 times for every one standard deviation increase (95% CI [1.36–1.69]) (Figure 3B).

### Subgroup analyses

**Figure 4:**
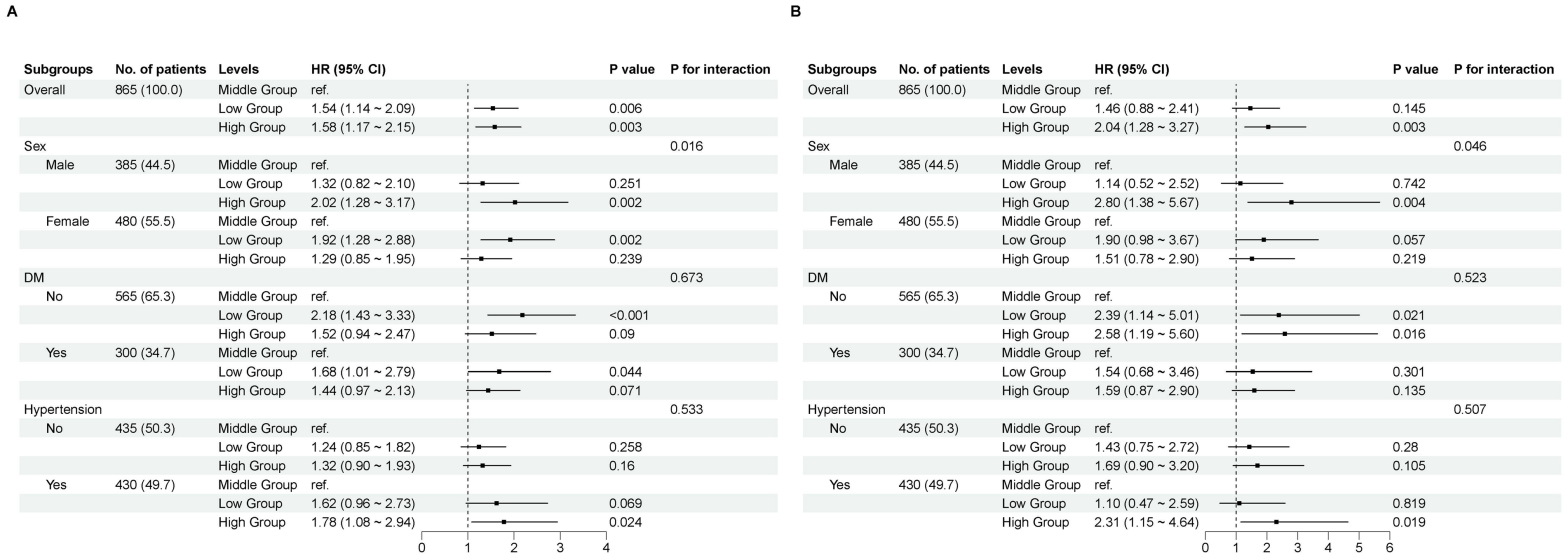
Subgroup analyses. A comparison of the adjusted hazard ratios for mortality. **(A)** All−cause mortality. **(B)** Cardiovascular mortality.

In the sensitivity analysis, our research findings remained robust, with the exception of sex as a factor. We observed that higher TyG–BMI values were associated with increased all-cause and cardiovascular mortality in men, whereas in women, lower TyG–BMI values were linked to elevated all-cause mortality but showed no difference in cardiovascular mortality risk between the groups (*p* = 0.016, *p* = 0.046). Therefore, adjustments for comorbidities such as hypertension and diabetes were not necessary (Figure 4).

## Discussion

Chronic kidney disease (CKD) has emerged as a significant public health concern, affecting approximately 10% of the global population (1). Peritoneal dialysis (PD) represents one of the primary alternative treatment modalities for patients diagnosed with end-stage renal disease. To improve the survival rate and quality of life for patients undergoing PD, as well as to minimize the incidence of complications, researchers have focused on investigating the failure of ultrafiltration in PD (30), developing a predictive model for the risk of PD-associated peritonitis (31), and addressing the diagnosis and treatment of encapsulated peritoneal sclerosis (32) along with other complications associated with this therapy. Despite advancements that have extended the survival duration of these patients, concerns regarding mortality risk persist.

In this study, we observed an independent association between higher or lower TyG–BMI values and an elevated risk of all-cause mortality among the population undergoing PD. A U-shaped relationship was identified between TyG–BMI and all-cause mortality, with the inflection point at TyG–BMI = 209.73. At this time, patients have the lowest all-cause mortality rate, whereas those above or below this threshold have increased mortality rates. A J-shaped relationship was found between TyG–BMI and CVD mortality, with the inflection point at TyG–BMI = 206.64. The risk of CVD mortality remains relatively stable until the TyG–BMI reaches 206.64, after which it begins to increase rapidly. Sex differences were observed between the groups. Higher TyG–BMI values were associated with increased all-cause and cardiovascular mortality in men, whereas in women, lower TyG–BMI values were linked to elevated all-cause mortality but showed no difference in cardiovascular mortality risk between the groups. However, comorbidities such as hypertension and diabetes did not influence these relationships. These findings support the potential clinical utility of the TyG–BMI as a reference value and predictive marker. It is also essential to consider the moderating impact of sex differences.

Numerous studies conducted on animals or humans have demonstrated that elevated triglyceride levels, impaired glucose metabolism, and increased BMI in CKD are linked to the activation of the renin–angiotensin system, the β-catenin signaling pathway, dysbiosis of the intestinal microbiota, abnormal long non-coding RNAs (LncRNAs), and various metabolic disorders. These factors heighten the risk of mortality (33–42). A number of publications have reported that elevated TyG–BMI is associated with an increased risk of cardiovascular diseases (19–22). Our findings similarly indicate that higher TyG–BMI values are associated with an elevated risk of all-cause and CVD mortality in individuals undergoing PD, compared to those with TyG–BMI values ranging between 189.57 and 227.15. This association may be attributed to the fact that participants with high TyG–BMI values presented higher SBP, TGs, and BMI, along with a higher incidence of diabetes and original diseases, such as diabetic nephropathy and hypertensive nephropathy, all of which contribute to increased mortality. Notably, IR was identified as one explanation for this association (43) and is a prominent characteristic in end-stage kidney disease patients (8, 14, 15, 44, 45), particularly in PD patients. In PD patients, prolonged exposure to glucose solutions, which are widely used in most countries, may lead to systemic hyperglycemia and obesity, while exacerbating IR due to peritoneal glucose exposure along with advanced glycation end products and bioincompatible solutions (16, 17). On the basis of this premise, IR can induce an imbalance in glucose metabolism, which subsequently triggers inflammation and oxidative stress. Furthermore, IR can stimulate the increased production of free radicals and glycosylated products, leading to nitric oxide (NO) inactivation (46, 47).

Kiran et al. elucidated a U-shaped correlation between BMI and mortality by enrolling 274 Asian PD patients (48). Similarly, our findings revealed that a lower TyG–BMI value was associated with an increased risk of all-cause mortality compared to intermediate TyG–BMI values, a relationship potentially linked to poor nutritional status (49, 50). The TyG–BMI exhibited a U-shaped association with all-cause mortality in the population undergoing PD. The level associated with the lowest risk of all-cause mortality ranged from 189.57 to 227.15. Our results indicated that higher and lower levels of glucose, triglycerides, or BMI could lead to poorer prognosis. These findings strongly support the need to establish target ranges for triglycerides, glucose, and BMI rather than specific target levels.

Numerous studies have demonstrated that the TyG–BMI serves as an independent predictor of adverse cardiovascular events (19–22). It is also closely associated with IR, which not only contributes to the development of CVD in both CKD patients and diabetic patients but also predicts the cardiovascular prognosis of CVD patients (14, 47). Previous research has consistently shown a significant correlation between TyG–BMI and future CVD mortality, myocardial infarction, and stroke, indicating that insulin resistance plays a pivotal role in the pathogenesis and prognosis of cardiovascular diseases (20, 22). Our study similarly revealed a significant association between TyG–BMI and cardiovascular mortality. When the two groups were compared, the high TyG–BMI group clearly presented significantly higher rates of CVD-related death than the middle group did across all three models; however, no significant difference was observed between the low TyG–BMI group and the middle TyG–BMI group.

Interestingly, we observed that sex had a modifying effect on the risk of mortality. This phenomenon may be attributed to hormonal disparities (51), insulin resistance, visceral adiposity, endothelial dysfunction, and chronic inflammation. Furthermore, lifestyle variations (e.g., a higher prevalence of risky health behaviors such as smoking, excessive alcohol consumption, and sedentary habits among men) contribute to increased all-cause and cardiovascular disease mortality in male patients with elevated TyG–BMIs. Naturally, this finding necessitates thorough examination and validation within a more extensive cohort of peritoneal dialysis patients.

### Study strengths and limitations

In this study, we identified an independent association between the TyG–BMI and all-cause as well as CVD mortality in a single PD center. Furthermore, we observed for the first time that the relationship between the TyG–BMI and all-cause mortality exhibited a U-shaped pattern in the population undergoing PD.

However, our study has several limitations. First, the levels of triglycerides and glucose may have been influenced by prescribed medications, which were not reported in our study, potentially introducing bias to the results. Second, data on triglycerides, glucose, and BMI were collected only once at baseline, and it remains unclear whether changes in TyG–BMI over time could impact its association with mortality. Therefore, longitudinal cohort studies are necessary to explore the persistence of the association between TyG–BMI and mortality over time. Third, we did not assess the homeostasis model assessment of insulin resistance or compare it with the TyG–BMI because of insufficient data on insulin levels during follow-up. Finally, potential residual confounding factors should be acknowledged, given that this is an observational study.

## Conclusion

The TyG–BMI demonstrated a U-shaped relationship with all-cause mortality and a J-shaped relationship with CVD mortality among patients undergoing PD. The inflection points were identified at 209.73 and 206.64. Additionally, significant sex differences were observed in these associations.

## Data Availability

The raw data supporting the conclusions of this article will be made available by the authors, without undue reservation.
